# Neuropsychological Functioning in Bilateral versus Unilateral Temporal Lobe Epilepsy

**DOI:** 10.3390/brainsci13111526

**Published:** 2023-10-29

**Authors:** Martina Baggio, Alberto Danieli, Cristiano Crescentini, Gian Marco Duma, Martina Da Rold, Sara Baldini, Eric Pascoli, Lisa Antoniazzi, Alec Vestri, Franco Fabbro, Paolo Bonanni

**Affiliations:** 1 Epilepsy and Clinical Neurophysiology Unit, Scientific Institute, IRCCS E. Medea, Via Costa Alta 37, 31015 Conegliano, Italy; martina.baggio@lanostrafamiglia.it (M.B.); alberto.danieli@lanostrafamiglia.it (A.D.); alec.vestri@lanostrafamiglia.it (A.V.); paolo.bonanni@lanostrafamiglia.it (P.B.); 2 Department of Languages and Literatures, Communication, Education and Society, University of Udine, 33100 Udine, Italy; 3 Aulss 2 Marca Trevigiana, Piazzale Dell’Ospedale, 1, 31100 Treviso, Italy; 4 Clinical Unit of Neurology, Department of Medical Sciences, University Hospital and Health Services of Trieste, University of Trieste, 34129 Trieste, Italy; 5 Department of Medicine—DAME, University of Udine, 33100 Udine, Italy; 6 PERCRO Perceptual Robotics Laboratory, Scuola Superiore Sant’Anna, 56010 Pisa, Italy

**Keywords:** temporal lobe epilepsy, neuropsychology, memory

## Abstract

Although relatively specific anatomo-electro-clinical features of temporal lobe epilepsy (TLE) with bilateral ictal involvement (bitemporal epilepsy—BTLE) have been described, differentiating between BTLE and unilateral TLE (UTLE) remains challenging. Surgery is often the treatment of choice for drug-resistant UTLE, whereas its use is more controversial in BTLE. It is currently unclear whether neuropsychological assessment can contribute to the differential diagnosis. We retrospectively reviewed the neuropsychological evaluation of 46 consecutive patients with refractory TLE. Eighteen patients were diagnosed with BTLE on the basis of ictal electro-clinical data, in particular a video EEG recording of at least one seizure simultaneously involving the two temporal lobes without the possibility of lateralizing its onset or at least two different seizures independently arising from the two temporal lobes. Twenty-eight patients were classified as UTLE. Presurgery evaluation data were used in this study. Compared with UTLE, BTLE was associated with a lower intelligence quotient (IQ) and more severe impairment in long-term memory, the latter remaining significant even after controlling for IQ. No significant differences were found between right and left UTLE. In conclusion, BTLE and UTLE are associated with relatively distinct neuropsychological profiles, further supporting their classification as different disorders within the TLE spectrum.

## 1. Introduction

Temporal lobe epilepsy (TLE), the most common form of medically refractory focal epilepsy, has been associated with impairments in multiple cognitive functions, particularly memory [1,2,3,4]. Recent studies indicate that neuropsychological deficits in patients with TLE tend to be either restricted to memory and/or language, or relatively widespread [5,6]. Several clinical variables, including age at epilepsy onset, seizure frequency (particularly generalized seizures), and disease duration have been associated, although not univocally, with an increased risk of cognitive impairment [7]. At the same time, advanced neuroimaging techniques have revealed a spectrum of (micro)structural and functional brain abnormalities involving cortical and subcortical structures, the extension and severity of which reflect the variability of cognitive phenotypes, at least partly independently from the presence of hippocampal sclerosis (HS) and other epileptogenic lesions [8,9,10]. Temporal lobe seizures can originate from mesial (limbic), lateral (neocortical), or both mesial and lateral structures [11,12,13]. Although mesial TLE is the most frequent and best characterized form of TLE, seizures with initial discharge involving lateral or both mesial and lateral structures are not infrequent [12]. Moreover, a proportion of patients with TLE may present with seizures involving both temporal lobes (bilateral temporal lobe epilepsy, BTLE) either independently, sequentially, or simultaneously [14,15]. Surgery is the treatment of choice for UTLE, particularly when clinical data and non-invasive investigations (including video EEG, MRI, and PET) are consistent with a clearly lateralized mesial temporal lobe epilepsy. Its use is more controversial in BTLE. On the one hand, relatively specific features of BTLE compared with UTLE have been reported, including a higher frequency of different seizure semiology and lateralizing phenomena, pointing to bilateral independent seizure onset [16], a lower frequency of ictal motor signs and longer duration of postictal unresponsiveness [17], more frequent auditory auras and bilateral upper limb dystonia, a more tardive epilepsy onset, more frequent bilateral asynchronous interictal EEG epileptiform discharges, and no MRI lesions other than hippocampal sclerosis [14]. On the other hand, it is currently unclear whether non-invasive electroclinical recordings reliably allow us to differentiate between UTLE and true BTLE. Case series of patients who underwent intracranial EEG (iEEG) after a presumed diagnosis of BTLE based on scalp ictal VEEG revealed that TLE was unilateral in a significant proportion of them [18]. Studies based on stereoelectroencephalography (SEEG) emphasized the importance not only of seizure lateralization but also of the network organization of the epileptogenic zone, whereby a mesial temporal lobe seizure pattern affecting the temporal lobes independently, with a good prognosis after unilateral anterior temporal lobectomy, could be differentiated from a more complex bitemporal multifocal mesial-lateral seizure network, a clear contraindication for surgery [15]. Overall, the current literature suggests that BTLE and UTLE can be differentiated based on anatomo-electro-clinical features, although at times only by means of invasive studies, and may represent different forms of epilepsy within the TLE spectrum. Whether the differences also extend to neuropsychological functioning is unclear. Given the challenges related to the differential diagnosis, the aim of this study was to analyze the neuropsychological profile of patients with BTLE and UTLE in order to identify possible further clinical differentiating features.

## 2. Materials and Methods

### 2.1. Participants

We retrospectively reviewed the clinical data of 52 consecutive adult patients with refractory TLE, who were admitted at Epilepsy and Clinical Neurophysiology Unit, E. Medea Scientific Institute in Conegliano between 2008 and 2018 for presurgical epilepsy evaluation. Each patient had undergone long-term scalp video EEG (VEEG) using a digital VEEG recording device (XLTEK or Micromed System Plus Evolution) with the electrodes placed according to the international 10–20 system and a neuropsychological assessment. Patients were selected based on the following inclusion criteria: (a) an assessment of overall intelligence with a standardized scale (i.e., the Italian version of the Wechsler Adult Intelligence Scale, WAIS-IV, or WAIS-R; [15,19]), with a reliable measurement of full-scale Intelligence Quotient (i.e., FSIQ > 45) and (b) a memory assessment with standardized neuropsychological tests. Six patients were excluded: three had FSIQ ≤ 45, (one UTLE and two BTLE); for the other three, either the FSIQ or memory assessment was not available. The final sample of patients thus included 46 individuals. BTLE was diagnosed based on the ictal electro-clinical criteria proposed by Didato et al. [14]: a VEEG recording of at least one seizure simultaneously or sequentially involving the two temporal lobes without the possibility of lateralizing its onset (non-lateralisable bitemporal seizure-NL), or the recording of at least two different seizures independently arising from the two temporal lobes (independent bitemporal seizure—IND). Eighteen patients (thirteen females, mean age: 42.02 ± 11.89 SD years, range: 21.5–62.4) met the criteria for BTLE. Twenty-eight patients were diagnosed with UTLE (nineteen females, mean age: 36.86 ± 10.77 SD years, range: 22.8–63), fifteen with right TLE (R-TLE) (ten females, mean age: 36.84 ± 11.72 SD years, range: 22.8–63) and thirteen with left TLE (L-TLE) (nine females, mean age: 36.87 ± 10.09 SD years, range: 23–51.11). Sixteen patients with UTLE (six males, mean age: 33.37 ± 8.9 SD) underwent epilepsy surgery, while the others were either waiting for surgery or had refused it. The clinical and demographic characteristics of the patients are reported in Table 1. During the presurgical assessment, a complete neuropsychological evaluation was performed, although with partially differing tests. For this retrospective study, we focused our analysis on memory function, given its relevance for TLE, and selected the tests available for the largest proportion of patients. The characteristics of the sample and the tests we focused on are reported in Table 1.

### 2.2. Cognitive and Neuropsychological Assessment

We reviewed measures of the global cognitive functioning, as well as data on specific cognitive functions such as memory and attention. In the paragraphs below, we provide a detailed representation of the neuropsychological data used in the analysis.

### 2.3. Global Cognitive Function

The Wechsler Adult Intelligence Scale (WAIS-R) scale and the Wechsler Adult Intelligence Scale IV were used as a measure of the global IQ [15,19].

### 2.4. Long-Term Memory

#### 2.4.1. Verbal Long-Term Memory

Verbal memory performance was assessed using a modified Italian version of the Rey Auditory Verbal Learning Test—R AVLT [20]. The RAVLT requires serial learning and immediate recall of 15 words in five consecutive learning trials, free recall after distraction, as well as free recall and recognition of the target words after a 15 min delay. Analysis was based on learning performance (total number of words learned in five trials) and delayed free recall.

#### 2.4.2. Visuo-Spatial Long-Term Memory

The ability to store visual information in long-term memory was assessed with the Rey Osterrieth Complex Figure (ROCF) [21]. A subject has to copy the complex figure and then, after 5 min, reproduce it from memory. The test is useful for evaluating neurological dysfunction in visual perception and long-term visual memory.

### 2.5. Short-Term Memory

#### 2.5.1. Verbal Short-Term Memory

Digit Span Test is a test of immediate memory [20,22]. The patient is asked to repeat a string of numbers which is made progressively longer in order to determine the maximum amount of numbers the patient can recall. In the normative population, the mean recall is around six or seven numbers. The final score is the number of correctly repeated digits.

#### 2.5.2. Visuo-Spatial Short-Term Memory

The Corsi block-tapping test [23] is a psychological test that assesses visuo-spatial short-term working memory. It involves mimicking a researcher as he/she taps a sequence of up to nine identical spatially separated blocks. The sequence starts out simple, usually using two blocks, but becomes more complex until the subject’s performance declines. This number is known as the Corsi Span, and averages about 5 for normal human subjects. When the sequence to be recalled becomes longer than three or four items, central executive resources are used.

### 2.6. Data Analysis

The scores obtained by each patient were expressed as scaled scores for all the analyzed intelligence and neuropsychological measures. For the FSIQ, digit span test, Corsi block tapping Test, ROCF (Copy and recall), RAVLT immediate recall, and RAVLT delay recall, we calculated them in relation to the normative values for the corresponding chronological ages. Normal distribution of demographic (age, years of education) and clinical (duration and onset of epilepsy, number of seizures per month) characteristics and neuropsychological features were verified for each group of patients (R-TLE, L-TLE, BTLE) using the Shapiro–Wilk W test (α value < 0.01 for the normality tests). Then, independent sample *t*-tests for normally distributed data and Mann–Whitney tests for not normally distributed data (number of seizures per month and the years of education) were performed to explore global differences between unilateral and bilateral patients with temporal lobe epilepsy (level of significance α = 0.05). In case of a significant difference between the two groups, one-way ANOVAs were run with the factor Group at three levels (R-TLE, L-TLE, and BTLE) to investigate whether a particular subgroup was responsible for the difference. Overall, we used Bonferroni’s method to correct for multiple comparisons, considering the number of outcome variables included in the neuropsychological domain of memory (i.e., 6 variables, α value < 0.008). Effect sizes were calculated to explore clinically meaningful differences between the different clinical groups in the memory measures. Cohen’s d was computed, and effect sizes were considered small (d = 0.20), moderate (d = 0.50), large (d = 0.80), and very large (d > 1). The data were analyzed with Statistica (version 8.0) (StatSoft, Inc., Tulsa, OK, USA).

## 3. Results

### 3.1. Participant Characteristics and Clinical Factors

The two groups of patients were matched for years of education (mean 11.79 ± SD 3.01 unilateral, mean 10.27 ± SD 2.90 bilateral, U =177, *p* = 0.07), age (mean 36.85 ± SD 10.77 unilateral, mean 42.02 ± SD 11.89 bilateral, t (45) = 1.53, *p* = 0.13), duration of epilepsy (mean 16.90 ± SD 11.46 unilateral, mean 24.32 ± SD 14.99 bilateral, t (44) = 1.89, *p* = 0.06), onset of epilepsy (mean 20.37 ± SD 12.56 unilateral, mean 17.70 ± SD 16.14 bilateral, t (44) = 0.62, *p* = 0.53), number of seizures per month (mean 13.35 ± SD 17.71 unilateral, mean 12.55 ± SD 17.70 bilateral, U = 250.5, *p* = 0.97), and gender (*p* = 0.75, Fisher’s exact test, two-tailed).

### 3.2. Intelligence and Neuropsychological Assessment

Table 2 reports the intelligence and neuropsychological (memory) outcomes of the UTLE and BTLE groups. The FSIQ score was significantly lower in the BTLE group compared with UTLE patients (bilateral < unilateral, *p* = 0.013; Table 2). Of note, a total of eight patients showed a mild or moderate intellectual disability (45 < FSIQ < 70; M = 59.87, SD = 6.49 for these eight patients). Patients with BTLE scored significantly lower than patients with UTLE in the verbal long-term memory tests (RAVLT later recall, *p* < 0.001 and RAVLT immediate recall, *p* = 0.003, Table 2), and more marginally in the visuo-spatial long-term memory tests (ROCF copy, *p* = 0.020 and ROCF reproduction, *p* = 0.019, Table 2). The difference between unilateral and bilateral patients in the two conditions in terms of verbal long-term memory appeared to be clinically relevant, eliciting large effect sizes (d = 1.47 and d = 1.15, respectively). No differences between unilateral and bilateral patients with epilepsy were found in the short-term memory measures the Digit Span test and Corsi block tapping test (*p* = 0.154; *p* = 0.338) (Table 2).

We further tested whether the effects found for the two conditions of the verbal long-term memory test (RAVLT delay recall, RAVLT immediate recall) could depend on a specific subgroup of patients with unilateral epilepsy (R-TLE or L-TLE). We ran, therefore, two one-way ANOVAs in which we tested the effect of the categorical independent variable “Group” (R-TLE, L-TLE, and BTLE) and “test condition” (delay recall, immediate recall). For the RAVLT delay recall condition, the analysis showed a significant effect of Group (F (2,29) = 8.62, *p* = 0.001; η_p_^2^ = 0.372). The post hoc tests (Duncan correction for multiple comparisons) showed that this effect was due to patients with BTLE, who performed worse in the verbal long-term memory test than both the patients with R-TLE (*p* < 0.001) and L-TLE (*p* = 0.014); no difference between R-TLE and L-TLE was found (*p* = 0.225). Similarly, the analysis concerning the RAVLT immediate recall test also showed a significant effect of Group (F (2,30) = 5.68, *p* = 0.008; η_p_^2^ = 0.274). The post hoc tests (Duncan correction for multiple comparisons) confirmed that these results depended on the worse performance of the BTLE patients compared with those with R-TLE (*p* = 0.005) and, more marginally, with L-TLE (*p* = 0.058); with no difference between R-TLE and L-TLE being found (*p* = 0.249).

Although we found only marginal effects for the two conditions in terms of visuo-spatial long-term memory (ROCF copy and reproduction), one-way ANOVAs (one for each visuo-spatial long-term memory condition) showed significant effects of Group (F (2,39) = 3.75, *p* = 0.03; η_p_^2^ = 0.161 for ROCF copy and F (2,38) = 3.18, *p* = 0.05; η_p_^2^ = 0.143 for ROCF reproduction). The post hoc tests showed that these effects were due to patients with BTLE performing worse on the visuo-spatial long-term memory tests than patients with L-TLE (both *p* < 0.05) but not with R-TLE (both *p* > 0.10), with no difference found between R-TLE and L-TLE (both *p* > 0.20). Finally, we considered whether the effects found for the two tests of verbal long-term memory (RAVLT immediate recall, RAVLT delayed recall) could depend on general intelligence, which was lower for bilateral than unilateral patients with temporal epilepsy. First, we performed two one-way ANOVAs (one for each memory test condition: immediate and delayed recall), in which we tested the effects of the independent variable of Group (unilateral, bilateral) and of the covariate FSIQ. In both ANOVAs, we found significant effects for the variable Group (RAVLT immediate recall F (1,30) = 7.20, *p* = 0.011, RAVLT delayed recall: F (1,29) = 12.41, *p* = 0.001) but not for FSIQ (RAVLT immediate recall: F (1,30) = 0.75, *p* = 0.391, RAVLT delayed recall, F (1,29) = 0.18, *p* = 0.67). To summarize, BTLE patients perform worse on the verbal long-term memory test (RAVLT immediate recall and delayed recall) than R-TLE and L-TLE patients. Additionally, patients with BTLE display a reduced performance also in the visuo-spatial long-term memory test (ROCF reproduction) when compared with L-TLE but not with R-TLE patients. This latter set of results was also confirmed when we repeated the initial independent sample *t*-tests on the two verbal long-term memory tasks excluding those patients with intellectual disability (45 < FSIQ < 70; 3 patients with UTLE and 5 patients with BTLE): (1) for RAVLT immediate recall, the results were as follows: unilateral: M = 43.53 ± 6.86, bilateral: M = 35.73 ± 9.16, t (26) = 2.57, *p* = 0.016; and (2) for RAVLT delayed recall, the results were as follows: mean 8.73, SD ± 2.78 unilateral, mean 4.61, SD ± 3.23 bilateral, t (25) = 3.48, *p* = 0.002. Once the eight patients with 45 < FSIQ < 70 were excluded from the analyses, the two groups of patients (unilateral, bilateral) showed similar FSIQ scores (mean 95.8, SD ± 13.69 unilateral, mean 87, SD ± 13.89 bilateral, t (37) = 1.88, *p* = 0.067). Importantly, in these inferential statistics, we did not include the three patients with the FSIQ ≤ 45, since the diffused cognitive impairment would have been generalized to multiple cognitive domains, making a comparison with the other patients not reliable.

To improve the clinical usefulness of our data, we wanted to highlight the meaning of the global intelligence quotient, considering for each clinical group the proportion of patients that scored under the clinical cut-off. For this analysis, we included the three patients with an IQ below 45 that were removed from the previous analyses. Then, we extended the same analysis to memory function. The results are reported in Table 3.

In Table 3, the BTLE patients showed a higher percentage of scores under the clinical cut-off compared with patients with UTLE. Intellectual disability was present in 35% of BTLE patients and 14% of UTLE. Deficits in verbal long-term memory were more common in L-TLE than R-TLE patients (22% vs. 10%), while deficits in visual long-term memory were more frequently observed in R-TLE than L-TLE patients (50% vs. 33%).

## 4. Discussion

Our study demonstrates that the differences between BTLE and UTLE extend to neuropsychological functioning. Compared with UTLE, BTLE was associated with lower overall IQ, an increased prevalence of intellectual disability (ID), and worse long-term memory. Notably, patients with BTLE showed a more severe impairment of long-term memory even after controlling for IQ, indicating that such findings cannot be primarily attributed to differences in general intelligence. Furthermore, the difference in verbal long-term memory between BTLE and UTLE (right and left) was stronger compared with the difference found in visual long-term memory. Several clinical variables have been associated with reduced performances in overall cognitive functioning and multiple neuropsychological domains, including age at epilepsy onset, disease duration, seizure frequency (particularly of generalized seizures), anti-seizure medications, education, and socio-economic status [8,24,25,26]. In recent years, significant correlations between neuropsychological functioning and structural and functional MRI abnormalities in TLE have also been reported. Hermann and colleagues [5] found a correlation between the prevalence and severity of neuropsychological impairments and the extent of MRI volumetric abnormalities involving extratemporal structures in adults with temporal lobe epilepsy. Additional findings documented a pronounced microstructural alteration of the superficial white matter involving the frontal and temporo-limbic regions ipsilateral to the seizure region in patients with UTLE, which correlated with verbal memory impairments [9]. Similarly, it has been demonstrated that different profiles of neuropsychological impairments are associated with distinct patterns of white matter microstructural abnormalities in TLE [8]. Further investigations identified specific functional and microstructural abnormalities affecting language networks in the subgroup of language-impaired patients with TLE, an imaging signature that was not related to epilepsy lateralization or other clinical variables except for age of onset [10]. Finally, Neal and colleagues [27] analyzed the preoperative resting state fMRI (RS-fMRI) of patients with TLE who had undergone surgical treatment and found that a higher connectivity within the hemisphere contralateral to the seizure onset zone was associated with higher rates of seizure recurrence and neuropsychological impairments. However, studies specifically investigating such correlations in BTLE are comparatively rare. Mirò and colleagues [28] documented larger temporal and extra-temporal white matter microstructural and volumetric abnormalities in BTLE with bilateral HS compared with UTLE [28]. The abnormalities in BTLE extended to the commissural pathways, possibly indicating a role of these structures in interhemispheric seizures propagation. The higher prevalence of cognitive deficits observed in BTLE compared with UTLE in our study may therefore reflect the presence of a more distributed epileptic network in the former group. This suggests that BTLE may involve a distinct etiopathology (“primary” BTLE) or may represent a different evolution of UTLE (“secondary” BTLE). Empirical evidence supporting either one or the other hypothesis is, however, limited, and the factors contributing to bilateral temporal epileptogenicity remain poorly understood. In our sample, most of the clinical variables potentially contributing to neuropsychological outcomes were comparable between UTLE and BTLE, except for epilepsy duration, which was higher in the BTLE group. Although this latter finding is consistent with the possibility that bilaterality in TLE could originate from a progressive, secondary spread of epileptogenesis, no differences regarding age at epilepsy onset and epilepsy duration between BLTE and TLE have been documented in other clinical cohorts [14,29,30]. Conversely, it has been suggested that BTLE may be characterized by a distinct anatomical and functional organization of the epileptogenic zone, with prominent involvement of the subhippocampal regions, which are more densely connected to the contralateral temporal regions [30]. Finally, in agreement with previous studies [14,29], the higher rates of “MRI-negative” patients in BTLE compared with UTLE (34% vs. 3%) observed in our sample further support the hypothesis of a partially distinct etiopathology between the two groups of disorders, possibly involving a proportion of patients with genetic factors of BTLE that are not associated with gross anatomical abnormalities. It is important to note that the diagnosis of BTLE in our study was based on non-invasive electroencephalographic recordings, limiting the possibility of defining the organization of epileptogenic networks involved in seizure generation and potentially leading to incorrect classification of UTLE as BTLE in some cases. A potential complementary approach to test the validity of our findings could be to retrospectively analyze the outcomes of SEEG and/or surgical treatment in patients with UTLE and BTLE in relation to presurgical neuropsychological functioning. At the same time, advanced techniques of EEG analysis, which allow us to investigate the functional organization of the brain, particularly the spatial and temporal properties of resting-state networks, may further contribute to the characterization of the relationship between the entity and distribution of functional abnormalities and the severity of cognitive impairment. High-density EEG has recently been applied to study resting-state dynamic network organization in patients with unilateral TLE, showing lower flexibility indices in the brain areas related to cognitive control and attention together with excessive internetwork communication, which were related to lower cognitive functioning [31]. This, as well as other EEG-based approaches, in particular microstates analysis (i.e., the analysis of the dynamic temporal organization of broad-band spontaneous EEG activity at rest) [32], may also improve our ability to differentiate between UTLE and BTLE by providing distinctive markers of the epilepsy-related brain network reconfiguration underlying these conditions.

## 5. Conclusions

In the present study, we compared for the first time the neuropsychological profiles of patients with BTLE and UTLE, showing that the former tend to present with a lower intelligence quotient and long-term memory. Our findings support the notion that UTLE and BTLE patients are relatively distinct anatomo-electro-clinical disorders within the spectrum of temporal lobe epilepsy [14]. Moreover, they indicate that neuropsychological testing can contribute to the differential diagnosis, thus improving patients’ classification and selection for surgical treatment. Prospective studies are needed to confirm our results and should ideally include other neuropsychological measures (e.g., attention, executive function, language), which have not been considered in the present study.

## Figures and Tables

**Table 1:** Demographic and clinical variables of patients with temporal lobe unilateral (either right, R-TLE, or left, L-TLE) and bilateral (IND-BTLE/NL-BTLE) epilepsy. Note. Pat: patient; yrs: years; SD: standard deviation; min: minimum; max: maximum; ASMs: anti-seizure medications; UTLE: unilateral temporal lobe epilepsy; F: female; R: right; L: left; LH: Left-handed; BTLE: bilateral temporal lobe epilepsy; IND: independent; NL: not lateralized.

	UTLE	BTLE
**Patients**	28	18
**Gender**	19F	13F
**Age, years (SD)**	36.85 (10.7)	42.02 (11.89)
**Epilepsy familiarity in Pat.**	4	2
**Years of Education (SD)**	11.8 (3.01)	10.27 (2.90)
**Handness**	6 LH	2 LH
**Type of TLE**	15 R-TLE	8 IND
	13 L-TLE	10 NL
**Age at onset, yrs (SD)**	20.37 (12.56)	17.60 (16.14)
**Epilepsy duration, yrs (SD)**	16.90 (11.46)	24.32 (14.99)
**Number of ASMs tried (min–max)**	4 (1–10)	5.5 (1–14)
**Number of ASMs at evaluation (min–max)**	1.8 (1–4)	2.6 (1–4)
**Seizures per month**	13.35 (17.71)	12.55 (17.70)

**Table 2:** Intelligence and neuropsychological memory performance of patients with temporal lobe unilateral (either right or left) and bilateral epilepsy. Notes: We analyzed the corrected score in relation to the normative values for the corresponding chronological ages for digit span test, Corsi block tapping test, and for RAVLT immediate recall and delayed recall and the z score for ROCF copy and reproduction. The t- and *p*-values in the table refer to the UTLE and BTLE comparison.

Test	UTLE Mean (SD)	L-TLE Mean (SD)	R-TLE Mean (SD)	BTLE Mean (SD)	t-Value	*p*-Value
Total IQ	92.34 (16.60)	98.46 (14.62)	86 (16.51)	79.05 (18.01)	2.58	0.013
Digit Span	5.31 (1.09)	5.20 (1.19)	5.33 (0.95)	4.82 (1.09)	1.45	0.154
Corsi Block Tapping	4.88 (0.97)	4.77 (1.26)	4.09 (0.86)	4.53 (1.33)	0.96	0.338
ROCFT Copy	0.04 (2.16)	0.78 (1.23)	−0.62 (2.56)	−2.23 (3.96)	2.42	0.020
ROCFT Reproduction	−1.10 (1.26)	−1.11 (1.28)	−1.26 (1.40)	−2.10 (1.30)	2.43	0.019
RAVLT immediate recall	43.04 (6.84)	40.48 (6.98)	44.97 (6.70)	34.11 (9.35)	3.17	0.003
RAVLT delayed recall	8.63(2.91)	7.20 (3.44)	9.4 (3.03)	4.39 (3.08)	3.95	<0.001

**Table 3:** Percentage of patients who scored under the clinic cut-off in tests of Total IQ and long- and short-term memory.

Neuropsy Tests	Clinical Cut-Off	N. Pat UTLE (29)	R-TLE (15)	L-TLE (14)	N. Pat BTLE (20)
Total IQ	<70	(4/29) 14%	(2/15) 13%	(2/14) 14%	(7/20) 35%
Digit Span	<3.5	(0/27) 0%	(0/15) 0%	(0/12) 0%	(2/19) 10.53
Corsi Block Tapping	<3.5	(2/24) 8.33%	(0/13) 0%	(2/11) 18%	(6/18) 33.33%
ROCFT Copy	Z > −1.5	(5/26) 19.23%	(4/14) 28.57%	(1/12) 8.3%	(11/18) 61.11%
ROCFT Reproduction	Z > −1.5	(15/26) 42.31%	(7/14) 50%	(4/12) 33%	(14/18) 77.78%
RAVLT immediate recall	<28.5	(0/19) 0%	(0/10) 0%	(0/9) 0%	(4/16) 25%
RAVLT delayed recall	<4.6	(3/19) 15.79%	(1/10) 10%	(2/9) 22%	(7/15) 46.67%

## Data Availability

The data that support the findings of this study are available on request from the corresponding author [M.B.]. The data are not publicly available due to privacy restrictions.
